# Genetic parameters of resistance to *Vibrio aestuarianus*, and OsHV-1 infections in the Pacific oyster, *Crassostrea gigas*, at three different life stages

**DOI:** 10.1186/s12711-017-0297-2

**Published:** 2017-02-15

**Authors:** Patrick Azéma, Jean-Baptiste Lamy, Pierre Boudry, Tristan Renault, Marie-Agnès Travers, Lionel Dégremont

**Affiliations:** 1 Laboratoire de Génétique et Pathologie des Mollusques Marins, Ifremer, avenue Mus de Loup, 17390 La Tremblade, France; 2 Laboratoire des Sciences de l’Environnement Marin, UMR 6539 LEMAR (UBO/CNRS/IRD/Ifremer), Centre de Bretagne, Ifremer, CS 10070, 29280 Plouzané, France; 3 Département Ressources Biologique et Environnement, Ifremer, Rue de l’Ile d’Yeu, 44300 Nantes, France

## Abstract

**Background:**

In France, two main diseases threaten Pacific oyster production. Since 2008, *Crassostrea gigas* spat have suffered massive losses due to the ostreid herpesvirus OsHV-1, and since 2012, significant mortalities in commercial-size adults have been related to infection by the bacterium *Vibrio aestuarianus*. The genetic basis for resistance to *V. aestuarianus* and OsHV-1 and the nature of the genetic correlation between these two traits were investigated by using 20 half-sib sire families, each containing two full-sib families. For each disease, controlled infectious challenges were conducted using naïve oysters that were 3 to 26 months old. In addition, siblings were tested under field, pond and raceway conditions to determine whether laboratory trials reflected mortality events that occur in the oyster industry.

**Results:**

First, we estimated the genetic basis of resistance to *V. aestuarianus* in *C. gigas*. Susceptibility to the infection was low for oysters in spat stage but increased with later life stages. Second, we confirmed a strong genetic basis of resistance to OsHV-1 infection at early stages and demonstrated that it was also strong at later stages. Most families had increased resistance to OsHV-1 infection from the spat to adult stages, while others consistently showed low or high mortality rates related to OsHV-1 infection, regardless of the life stage. Our third main finding was the absence of genetic correlations between resistance to OsHV-1 infection and resistance to *V. aestuarianus* infection.

**Conclusions:**

Selective breeding to enhance resistance to OsHV-1 infection could be achieved through selective breeding at early stages and would not affect resistance to *V. aestuarianus* infection. However, our results suggest that the potential to select for improved resistance to *V. aestuarianus* is lower. Selection for dual resistance to OsHV-1 and *V. aestuarianus* infection in *C. gigas* might reduce the impact of these two major diseases by selecting families that have the highest breeding values for resistance to both diseases.

**Electronic supplementary material:**

The online version of this article (doi:10.1186/s12711-017-0297-2) contains supplementary material, which is available to authorized users.

## Background

The Pacific oyster, *Crassostrea gigas*, is the main oyster species cultivated worldwide, accounting for 98% of the global oyster production. However, oyster production is highly vulnerable to degradation of the environment by pollutants and infectious disease outbreaks. Mass mortality events in Pacific oysters have been reported since the 1950s in most producing areas, including Japan, the USA, Europe, New Zealand, and Australia. Different pathogenic agents (bacteria, viruses, and parasites) have been implicated in these outbreaks and can affect different life stages. One striking example concerns the oyster production in France, which is the fourth largest oyster producer in the world with an annual production of 79,000 tons of *C. gigas* for a value of 479.5 million US dollars in 2013 [1]. Originally, the French oyster production was based on (1) the flat oyster *Ostrea edulis*, but it decreased because of two protozoan parasites, *Bonamia ostreae* and *Marteilia refringens* [2], and (2) the Portuguese oyster *Crassostrea angulata*, which initially originated from Asia [3] but its production collapsed due to the detection of irido-like viruses [4]. Then, another exotic species, *C. gigas*, was massively introduced from Japan and British Columbia during the 1970s to sustain the French production [5]. Although, since its introduction, several pathogens have been detected in *C. gigas* during mortality outbreaks in France [6, 7], since 2008 such outbreaks have greatly risen at the spat (or seed) stage, due to a variant of the herpesvirus OsHV-1, named µVar [8–10], and since 2012 in adults, due to the bacterium *Vibrio aestuarianus* [11–13]. These two pathogens had already been detected in earlier mortality outbreaks [9, 14], but their impacts on French oyster production have been particularly significant since 2008.

Many factors may influence the dissemination of a disease, in particular, rearing practices, such as increased stocking densities on farms or transfer of infected animals [15]. Other factors may favor the vulnerability of the host such as stressful environmental conditions, including seawater pollution, acidification, and global warming, or the physiological status, including energy allocation for metabolic reserves during growth, gametogenesis, or spawning [16, 17]. Finally, the host immune competence for defense against a pathogen can be affected by environmental or genetic factors and genetic variability could be used in breeding programs to enhance disease resistance through a straightforward quantitative genetic approach [18, 19].

To date, selective breeding to improve oyster performance for the industry has mainly focused on traits such as growth [20–23], yield [24], or shell pigmentation [25]. Similarly, genetic improvement for disease resistance, which is considered as a promising approach to reduce disease losses in oyster production, has been successfully implemented in several countries [19]. Nevertheless, most studies in the literature did not provide estimated heritabilities or genetic correlations for disease resistance.

Recently, heritability for resistance to OsHV-1 infection was estimated in young (i.e. less than one year old) oysters, but not in larvae or adults [19]. Mortality events due to OsHV-1 infection were usually observed in spat (<2 cm/<5 g) or juvenile (2–5 cm/5–20 g) stages [17, 26–28]; adult oysters (>5 cm/>20 g) appear to be less susceptible to OsHV-1, both in field and laboratory trials [29, 30]. Recent experiments revealed that resistance to OsHV-1 infection increased with life stage [31, 32], but genetic parameters were not estimated in adults. To date, genetic parameters related to *V. aestuarianus* resistance/susceptibility have not been evaluated under field or controlled conditions, although differences in susceptibility to this bacterium between *C. gigas* stocks was reported [33, 34].

In the current study, two approaches were used to assess genetic resistance to *V. aestuarianus* and OsHV-1 infection in *C*. *gigas* during three successive life stages (spat, juveniles, and adults). In the first approach, experimental infections under controlled conditions in the laboratory were undertaken to estimate genetic parameters by minimizing environmental variations and for a single pathogenic agent. In the second approach, we carried out field trials in which oysters were faced to an uncontrolled environment with exposure to various pathogens. The field trials allowed us to determine whether the laboratory trials reflected the mortality events that are encountered by the oyster industry and which may result from a combination of several pathogens.

The objectives of our study were: (1) to investigate the susceptibility to *V. aestuarianus* and OsHV-1 infections at successive life stages; (2) to estimate the heritability of resistance to these infections at each life stage; and (3) to determine the genetic correlations between resistance to *V. aestuarianus* and OsHV-1 infections during a given life stage and between life stages.

## Methods

### Production of oyster families

In December 2012, *C. gigas* individuals were sampled from a wild population that was located at La Tremblade (Charente-Maritime, France), in the estuary of the Seudre River (Marennes-Oleron Bay: 45°46′53.5″N, 1°7′19.5″W). Oysters were brought to the Ifremer facilities in La Tremblade and placed in 240 L conditioning tanks. Seawater temperature was gradually increased to 21 °C during 1 week. Seawater flow was 400 L/h, and a cultured phytoplankton diet (*Isochrysis galbana*, *Tetraselmis suecica*, and *Skeletonema costatum*) was provided ad libitum (50,000 cells/mL). In March 2013, oysters were opened and sexed by microscopic observation of gonad samples spread on a slide. Gametes were collected by stripping the gonads from 20 sires and 40 dams. Eggs were sieved on 60-µm and then 20-µm mesh screens to remove large and small tissue debris, respectively and sperm was sieved on 60-µm mesh screens.

Fertilization was conducted following a nested half-sib design: each sire mated with two dams, producing 20 half-sib sire families (HSF), each containing two full-sib families (FSF). The 40 FSF were reared separately in 30 L tanks at 25 °C using UV-treated (40 mj/cm^2^) and filtered seawater (5 µm). Water was changed three times a week. Larvae were fed daily with *I. galbana* (30,000 cells/mL) until they reached 140 µm, and then the phytoplankton diet was complemented with *S. costatum* (30,000 cells/mL).

**Fig. 1:**
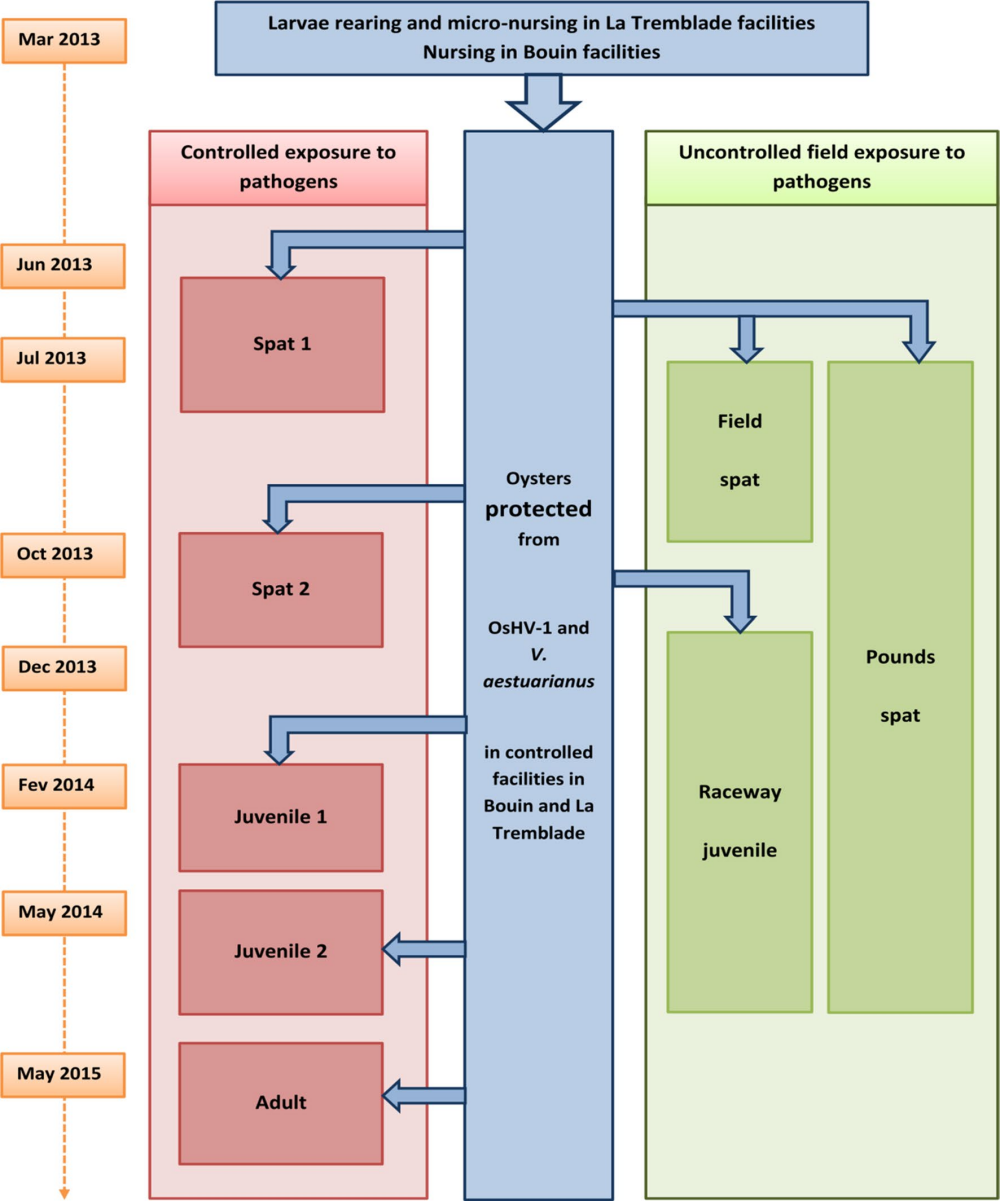
Experimental design

Fourteen to 18 days after fertilization, larvae from all crosses settled on cultch in 120 L raceways, which each contained six to seven separate families. Oyster seeds were reared under standard hatchery conditions until they reached a size of 2 mm. Then, in May 2013, 5000 oysters per family were transferred to the Ifremer nursery in Bouin (Vendée, France), while 2000 oysters per family remained in the hatchery facilities in La Tremblade. Oysters were kept in experimental facilities from spawning in March 2013 to May 2015 with UV-treated and filtered seawater and under biosecurity control to avoid contamination with OsHV-1 and *V. aestuarianus*. Each FSF was sampled 13 times during this period for genetic evaluation using either ten experimental infections under laboratory conditions (five ages × two pathogens) or three uncontrolled disease exposures in three environments that are commonly used by oyster farmers (field, pond, and raceways) (Fig. 1).

Three to 26-month old oysters were monitored in our study (Table 1). Three age groups were defined: spat (3 to 6 months old, <2 cm/<5 g), juvenile (11 to 15 months old, 2–5 cm/5–20 g) and adult (25 to 26 months old, >5 cm/>20 g).

**Table 1:** Summary of the trials to evaluate resistance to *V. aestuarianus* and OsHV-1 infections

	Controlled disease exposure in laboratory conditions										Uncontrolled disease exposure		
Experiment	Spat 1	Spat 2	Juv 1	Juv 2	Adult	Spat 1	Spat 2	Juv 1	Juv 2	Adult	Spat	Spat	Juvenile
Pathogen	*V. a.*	*V. a.*	*V. a.*	*V. a.*	*V. a.*	OsHV-1	OsHV-1	OsHV-1	OsHV-1	OsHV-1	Field	Ponds	Raceways
Date of challenge	July 2013	October 2013	March 2014	June 2014	June 2015	July 2013	October 2013	April 2014	July 2014	May 2015	July 2013–September 2013	July 2013–February 2014	December 2013–May 2014
Age (months)	3	6	11	14	26	3	6	12	15	25	3	3	8
Mean individual weights (g)	0.5	2.3	6.4	12.0	34.7	0.5	2.3	6.2	11.1	37.1	0.5	0.5	8.2
Number of tanks	1	1	3	3	6	1	1	3	3	2	1	1	3
Family/tank	40	40	40	40	39	40	40	40	40	39	40	40	40
Animal/family	75	75	45	60	30	75	75	45	60	10	100	100	170
Total number of oysters tested	3000	3000	1800	2400	1170	3000	3000	1800	2400	390	4000	4000	6800
Mean mortality (range among families) (%)	5 (0–11)	15 (0–38)	49 (20–92)	44 (24–72)	89 (67–100)	86 (21–100)	74 (23–100)	59 (7–100)	41 (8–83)	55 (0–100)	89 (25–100)	56 (40–75)	(62–100)
*V. aestuarianus* DNA detection in moribund	21/21	37/37	40/40	75/75	42/42	0/10	0/20	13/16	28/54	0/24	0/12	0/20	54/54
Mean number of copies/mL for 25 ng of total DNA	4.10E+04	2.00E+07	2.70E+07	5.60E+05	4.40E+07			2.00E+05	4.50E+02	.			4.00E+06
OsHV-1 DNA detection in moribund	0/21	0/37	0/40	12/75	10/42	10/10	20/20	16/16	54/54	23/24	12/12	0/20	16/54
Mean number of copies/µL for 25 ng of total DNA				7.00E+02	2.50E+03	2.50E+10	1.10E+07	1.70E+10	7.40E+07	7.30E+08	4.80E+09	.	6.90E+01

*V. a.*, *V. aestuarianus*; Juv 1 and 2, Juvenile 1 and 2; Spat, oyster <2 cm/<5 g; Juvenile, oyster 2–5 cm/5–20 g; Adult, oyster >5 cm/>20 g

No ethical approval was necessary for this study because experimental research on Pacific oyster, which is a bivalve mollusk, does not require special authorization.

### Experimental infections under controlled conditions

#### Bacterial and viral infectious suspensions

The *Vibrio* strain used in the bacterial challenges was the highly pathogenic strain 02/041, which was isolated during a mortality episode that affected adult oysters, and already studied [35]. This *Vibrio* suspension was obtained from an isolate maintained at −80 °C. Bacteria were grown in Zobell media and incubated for 24 h at 20 °C under constant agitation at 20 rpm. The resulting suspension was centrifuged at 3200*g* for 10 min. The supernatant was discarded and the pellet was washed and suspended in sterile artificial sea water (SASW).

The viral suspension was produced using the protocol described in [36]. Briefly, hatchery-produced oysters, which are considered as non-infected, were infected by injecting 50 µL of a viral suspension after “anesthesia” [37]. Experimentally-injected dead oysters were dissected to separate the mantle and gills, which were pooled, diluted, crushed and filtered on a 0.22-µm mesh to obtain a clarified tissue homogenate.

#### Genetic evaluation of resistance to OsHV-1 and *V. aestuarianus* infections

FSF were evaluated for resistance to OsHV-1 and *V. aestuarianus* infections under controlled laboratory conditions with five experiments for each pathogen: two experiments for the spat stage (Spat 1 and Spat 2) and two experiments for the juvenile stage (Juvenile 1 and Juvenile 2), and one experiment for adults (Adult) (Table 1). For each experiment, naïve oysters were tested.

Challenges under controlled conditions were performed in 100 L tanks with filtered (50 µm) and UV-treated (40 mj/cm^2^) seawater that was maintained at 21 °C, with aeration but without feeding, and 32‰ salinity. A recirculating system was used to optimize horizontal transmission of the infectious diseases by continuously homogenizing the seawater. All effluents were collected and treated by a specialized company to prevent introducing the pathogens used in the experimental infections into the environment.

Cohabitation disease challenge protocols, as previously described [38, 39], were adapted to evaluate disease resistance in *C. gigas*. Such protocols include two steps: (1) naïve oysters were anaesthetized in a solution containing magnesium chloride (MgCl_2_, 50 g/L) in a mixture of seawater and distilled water (1:4, v:v) for 4 h [37]; then, 50 µL of infectious bacterial (corresponding to 10^7^ bacteria/oyster) or viral suspensions (corresponding to 10^6^ viral copies/oyster) were injected into the adductor muscle using a 1-mL micro-syringe equipped with an 18-G needle; the injected oysters were transferred into 10 L tanks for 24 h; and (2), the oysters were placed in the same tank for 48 h with the 40 FSF to test disease resistance. Control tanks were added in each challenge protocol and included oysters that had been injected with SASW and placed in the same tank than the FSF. A ratio of 100 g of injected oysters to 10 L of seawater was used for all the experiments. All injected oysters were removed 48-h post-infection.

For *Vibrio* challenges, we used oysters that were 3, 6, 11, 14 or 26 months old with an average weight of 0.5, 2.3, 6.4, 12.0 and 34.7 g, respectively. For OsHV-1 challenges, we used oysters that were 3, 6, 12, 15 and 25 months old with mean individual weights of 0.5, 2.3, 6.2, 11.1 and 37.1 g, respectively (Table 1).

For the two experimental infections at the spat stage, one 100 L tank was used per disease that contained three soft mesh nets of 25 oysters per family, representing a total of 3000 oysters in the tank for a total biomass of 1.5 and 6.9 kg for Spat 1 and Spat 2, respectively. For the two juvenile stage experiments, three 100 L tanks were used for each disease to maintain a biomass under 10 kg per tank. Each tank contained the 40 FSF with 15–20 juveniles per family, representing 600 and 800 oysters, for a total biomass of 3.8 and 8.9 kg for Juvenile 1 and Juvenile 2, respectively. At the adult stage, the focus was on bacterial rather than viral infections, due to limited availability of oysters and a lack of data in the literature on resistance to *V. aestuarianus* in *C. gigas*. Thus, two and six 100 L tanks were used for the experimental infection with OsHV-1 and *V. aestuarianus*, respectively. Each tank contained 39 FSF (one FSF did not include a sufficiently large number of oysters to perform all the experiments because of a lower hatching rate), each represented by five individually-tagged oysters, for a total of 195 oysters and a biomass ranging from 6.7 to 7.2 kg. The tag was fixed on the upper shell with epoxy glue. Before genetic evaluation of the families at the adult stage, most of the oysters spawned.

Mortality was checked on a daily basis by observing the oysters in the tank. Mortality was estimated by counting the live and dead oysters at 5 and 11 days post-exposure for both disease exposures. An oyster was considered as dead when it was unable to close its valves after a 5 min-period out of the water.

### Testing spat under field and pond conditions

FSF oysters at the spat stage were deployed in the field at Agnas in the Marennes-Oléron Bay (45°52′23″N, 1°10′15″W) and in ponds called “claires,” at La Floride in La Tremblade (45°48′01″N, 1°09′09″W) on the 27th of July 2013. At deployment, oysters were 3 months old with an average weight of 0.5 g (Table 1). For each site, four bags were used, each containing the 40 FSF with 25 individuals per family, corresponding to 1000 individuals and 0.5 kg per bag. Within each bag, the families were separated using soft mesh bags with a label indicating the family name. For the experiment carried out in the pond, bags were fixed on racks, which were always immersed. The pond had an average depth of approximately 70 cm for a volume of 250 m^3^, and it was naturally supplemented with seawater during the spring tides, when the tidal coefficient exceeds 85. Survival was recorded each month from August 2013 to February 2014. For the field experiment, bags were fixed on racks, which emerged when the tide coefficient was above 70. Mortality was checked at 2 weeks post-planting and recorded at 6 weeks post-planting on the 6th of September. Seawater temperature was recorded hourly in each environment using two ThermoTrack probes (Progesplus, 59780, Willems, France).

### Testing juveniles under the raceway conditions

In December 2013, oysters from the nursery in Bouin were transferred into a pond in La Tremblade for 2 months. This pond was located near the one used for the pond conditions described above. Seawater temperature was below 10 °C in the pond and no mortality was observed during this period. In February 2014, oysters were placed in three 800 L flow-through raceways, filled with filtered and UV-treated seawater, at the Ifremer facilities in La Tremblade. For two of these flow-through raceways, 50 animals per family divided into two tagged soft mesh bags were tested in each raceway. For the third raceway, one tagged soft mesh bag was used per family, each containing 70 oysters. The initial aim of this testing was to study the occurrence of infectious diseases under confined conditions. Survival was recorded twice a month from February to May 2014. Seawater temperature was recorded hourly using two ThermoTrack probes (Progesplus, 59780, Willems, France). For control measures, siblings were not transferred into the pond, but directly placed in raceways in December 2013.

### Detection of OsHV-1 and *V. aestuarianus* DNA

In each experiment, the moribund oysters were sampled for detection of *V. aestuarianus* and OsHV-1 DNA (Table 1). Total DNA was extracted from tissue fragments (mantle and gills) using the QIAgen (Hilden, Germany) QIAamp tissue mini kit combined with the use of the QIAcube automate, according to the manufacturer’s protocol. The amount of total DNA was adjusted to 5 ng/µL after quantification with a Nanodrop instrument (Thermo Scientific).

A real-time PCR assay was conducted on the MX3000 and MX3005 thermocyclers (Agilent) using the Brilliant III Ultrafast kit (Stratagene). Each reaction was run in duplicate in a final volume of 20 µL containing the DNA sample (5 µL at 5 ng/µL), 200 nM of each primer (for OsHV1-µvar: DPF 5′ ATT GAT GATGTG GAT AAT CTG TG 3′ and DPR 5′ GGT AAA TAC CAT TGG TCT TGTTCC 3′ [40]; for *V. aestuarianus*: DNAj-F 5′ GTATGAAATTTTAACTGACCCACAA 3′ and DNAj-R 5′ CAATTTCTTTCGAACAACCAC 3′ [41]), and 200 nM of oligonucleotide probe (for *V. aestuarianus* DNAj: 5′ TGGTAGCGCAGACTTCGGCGAC). Real-time PCR cycling conditions were as follows: 3 min at 95 °C, followed by 40 cycles of amplification at 95 °C for 5 s, 60 °C for 20 s. For quantification of OsHV-1 DNA, melting curves were also plotted (55–95 °C) to ensure that a single PCR product was amplified for each set of primers. Negative controls (without DNA) were included.

### Statistical and quantitative genetic analyses

For all trials, statistical analyses of the recorded traits were performed with a cross-sectional model that analyzed survival at a fixed point in time. For both controlled (i.e. laboratory conditions) and uncontrolled disease exposures (field, pond and raceways), the status of each individual was recorded as a binary trait (dead = 0 and alive = 1). For disease exposure under laboratory conditions, mortality was analyzed at day 11 post-infection. For the field trial, mortality was recorded once at 6 weeks post-planting, and analyzed at this time. For pond and raceway experiments, mortality was recorded 8 to 19 times. We analyzed the mortality at day 146 for the pond experiment and at day 70 for the raceway experiment, when the mean mortality rate reached approximately 50%, at which point the phenotypic variance of a binary trait is maximized [42].

Genetic analyses were performed with a generalized linear mixed model using the Glimmix procedure of SAS software [43] and ASReml [44] using the following equation:

$${\mathbf{y}} = g^{ - 1} \left({\mathbf{X}\varvec{\upbeta}} + {\mathbf{Z}\varvec{\upgamma}}\right),$$

where **y** is the vector of $n$ observations (the binary trait dead = 0 and alive = 1 in our case), and $g^{ - 1} \left( \cdot \right)$ is the inverse of the logit function $\left( {\frac{{e^{\varvec{\eta}} }}{{{{\bf 1}} + e^{\varvec{\eta}} }}} \right).$

**X** is a ($n \times p$) design matrix of rank $k$ that links the observations to the vector ${\varvec{\upbeta }}$ of the $p$ fixed effects. Here, we considered three fixed effects: intercept, blocks (tanks or bags) and family average individual weight. Depending on the analysis, other fixed effects were added: life-stages (five life stages: Spat 1, Spat 2, Juvenile 1, Juvenile 2, and Adult) or pathogen treatments (OsHV-1 or *V. aestuarinaus*) (see below). **Z** is a ($n \times r$) design matrix that links the observations to vector ${\varvec{\upgamma }}$ of the *r* random effects. We did not constrain (co)variances matrices to be definitive positives (!GP qualifiers in ARSeml) since negative variances estimates are expected in case of small amounts of data [45]. As the mating plan was a North Carolina design I, we used a sire and dam model (estimations are similar to those in the animal model but the required computational time is much shorter and convergence more regular). Indeed, we considered two random effects: sire and dam (nested within sire) [45]. The random effects were assumed to be normally distributed as $N \sim \left( {0, {\mathbf{G}}} \right).$ The estimates of the generalized linear mixed model were given in the underlying liability scale and genetic parameters could be computed directly. Dominance was assumed to be negligible.

Across the three life stages, 11,370 and 10,590 oysters were phenotyped for *V. aestuarianus* and OsHV-1, respectively. To obtain meaningful estimates of heritability and genetic correlations between life stages, we analyzed simultaneously all experimental challenges for each disease using the model described above by setting-up the **G** matrix as follows:

$$\left[ {\begin{array}{*{20}l} {\sigma_{s}^{2} \otimes {\varvec{\Sigma}}_{j} } \hfill & 0 \hfill \\ 0 \hfill & {\sigma_{d}^{2} \otimes {\varvec{\Sigma}}_{j} } \hfill \\ \end{array} } \right],$$

where ${\varvec{\Sigma}}$ is an unstructured (co)variance square matrix with rank $j$ (five life stages: Spat 1, Spat 2, Juvenile 1, Juvenile 2, and Adult), $\sigma_{s}^{2}$ and $\sigma_{d}^{2}$ are the sire and dam variances, respectively within rank *j*, and the following fixed effects were included: life stage, blocks, and family average individual weight.

To estimate the genetic correlation between diseases (within or between each life stage), we used a bivariate model with a **G** matrix that was similar to that shown above except that ${\varvec{\Sigma}}$ is a square matrix with rank 2 (two traits) and the fixed effects were pathogen treatments, blocks and family average individual weight.

Both programs gave similar estimations (see Additional file 1: Figure S1), but since reaching convergence is more difficult with SAS when several random effects are considered with the pseudo-likelihood techniques, we only reported ASReml estimates.

Heritability of survival in each experiment was computed for the narrow sense $h_{n}^{2} ,$ as follows:

$$h_{n}^{2} = \frac{{V_{a} }}{{V_{t} }} = \frac{{4 \cdot \sigma_{m}^{2} }}{{\sigma_{m}^{2} + \sigma_{f}^{2} + \sigma_{e}^{2} }},$$

where $V_{a}$ is the additive genetic variance, estimated as four times the variance among sires $(\sigma_{m}^{2} )$ and $V_{t}$ is the total phenotypic variance, with $\sigma_{e}^{2}$ being $\frac{{\pi^{2} }}{3}$ for a logit link [46]. $\sigma_{f}^{2}$ is the variance among dams.

In addition, we also calculated genetic correlations of survival between the field experiment and the experiments under laboratory conditions, as well as between the raceway experiment and the experiments under laboratory conditions using bivariate models.

Estimated heritabilities and genetic correlations were significantly different from 0 when *P* < 0.05.

## Results

### Effects of life stage on susceptibility to *V. aestuarianus* and OsHV-1 infections

There was no mortality in the control tanks for all the experiments conducted in the laboratory.

**Fig. 2:**
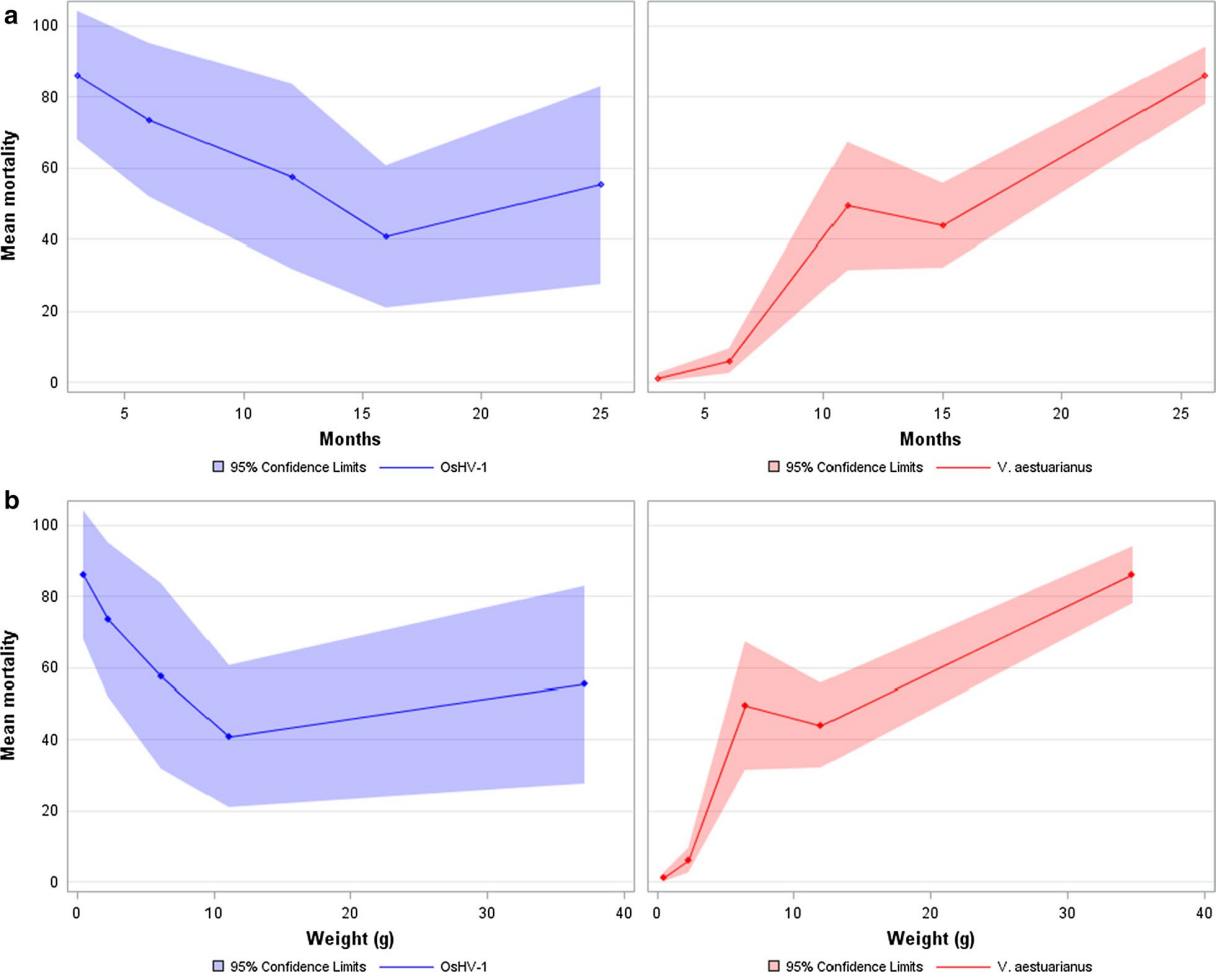
Mortality (%) at day 11 post-infection for OsHV-1 challenge (in *blue*) and for *V. aestuarianus* challenge (in *red*) under laboratory conditions. Band plots represent 95% confidence limits for all FSF, *dots* from the *left* to the *right* are for Spat 1, Spat 2, Juvenile 1, Juvenile 2 and Adult, respectively. **a** Evolution of sensibilities according to the age of the animals, **b** evolution of sensibilities according to the mean weight of animals

For *V. aestuarianus* infection, mortality was first observed at day 5 post-infection. At day 11, the mean mortality of the 40 FSF was low in Spat 1 (5%, ranging from 0 to 11%) and Spat 2 (15%, ranging from 0 to 38%), moderate in Juvenile 1 (49.3%, ranging from 20 to 91.9%) and Juvenile 2 (43.8%, ranging from 24.2 to 71.7%), and high in Adult (89%, ranging from 66.7 to 100%) (see Table 1; Fig. 2). Large numbers of DNA copies of *V. aestuarianus*, ranging from 4.1 × 10^4^ to 4.4 × 10^7^ copies/mL for 25 ng of total DNA, were found in all moribund oysters sampled from spat to adult stages (Table 1). In contrast, OsHV-1 DNA was not detected in oysters in Spat 1, Spat 2, or Juvenile 1, while 16 and 24% of the samples were positive in Juvenile 2 and Adult, respectively, at a low level (<10^3^ copies/µL for 25 ng of total DNA) (Table 1). Effect of life stage was highly significant followed by block and family average weight (all with *P* > 0.001).

Onset of mortality occurred at day 2 post-infection with OsHV-1 and reached a peak at day 5. At day 11, the mean mortality rate of the 40 FSF was high in Spat 1 (86%), ranging from 20.5 to 100%, and Spat 2 (73.5%) ranging from 23.5 to 100%. Mortality rate was lower in Juvenile 1 (58.6%, ranging from 6.7 to 100%), Juvenile 2 (40.7%, ranging from 8.3 to 83.3%), and Adult (54.9%, ranging from 0 to 100%) (see Table 1; Fig. 2). A large number of OsHV-1 DNA copies, ranging from 1.1 × 10^7^ to 2.5 × 10^10^ copies/µL for 25 ng of total DNA, was determined for all moribund oysters that were sampled from the spat to adult stage, except for one sample in Adult (Table 1). In contrast, *V. aestuarianus* DNA was not detected in Spat 1, Spat 2, or Adult, while 81 and 52% of the samples were positive with a high (2.0 × 10^5^ copies/mL for 25 ng of total DNA) and low (4.5 × 10^2^ copies/mL for 25 ng of total DNA) load in Juveniles 1 and Juveniles 2, respectively (Table 1). The effect of life stage was highly significant followed by block (all *P* > 0.001), family average weight was not significant (*P* = 0.49).

### Genetic parameters for resistance to *V. aestuarianus* infection differ between life stages whereas those for resistance to OsHV-1 infection are stable from spat to adult stages

Narrow sense heritabilities for survival of *C. gigas,* when exposed to *V. aestuarianus*, were low for both challenges at spat stages (0.09 ± 0.10 and 0.11 ± 0.07 at 3 and 6 months old, respectively) and moderate at the juvenile stages (0.26 ± 0.17 and 0.16 ± 0.09 at 11 and 15 months old, respectively) and the adult stage (0.33 ± 0.25 at 26 months old) (Table 2). Variance components are in Additional file 2: Table S1. Most of the genetic correlations between life stages, when exposed to *V. aestuarianus*, were positive, but they were significantly different from 0 only between Adult and Juvenile 2 or Spat 2, and between Juvenile 1 and Juvenile 2 (Table 2).

**Table 2:** Narrow sense heritability (diagonal) and genetic correlations (below diagonal) for survival of *C. gigas* when exposed to *V. aestuarianus* under laboratory conditions

	*V. aestuarianus*				
	Spat 1	Spat 2	Juvenile 1	Juvenile 2	Adult
*V. aestuarianus*					
Spat 1	0.09 ± 0.10				
Spat 2	0.55 ± 0.59	0.11 ± 0.07			
Juvenile 1	−0.25 ± 0.65	0.22 ± 0.47	0.26 ± 0.17		
Juvenile 2	0.34 ± 0.52	0.55 ± 0.30	0.99 ± 0.23*	0.16 ± 0.09	
Adult	0.66 ± 0.76	1.48 ± 0.60*	0.48 ± 0.48	1.10 ± 0.42*	0.33 ± 0.25

Spat, oyster <2 cm/<5 g; Juvenile, oyster 2–5 cm/5–20 g; Adult, oyster >5 cm/>20 g

* Significantly different from 0

Narrow sense heritabilities of survival of *C. gigas* to OsHV-1 infection were high: 0.99 ± 0.39 and 0.65 ± 0.31 for challenges in Spat 1 and Spat 2, respectively; 0.78 ± 0.33 in Juvenile 1; and 0.78 ± 0.49 in Adult. Narrow sense heritabilities were lower for Juvenile 2 i.e. 0.40 ± 0.21 (Table 3). Variance components are in Additional file 3: Table S2. Genetic correlations of survival between OsHV-1 challenges at different life stages were all high and positive, ranging from 0.55 to 1.06 (Table 3).

**Table 3:** Narrow sense heritability (diagonal) and genetic correlations (below diagonal) for survival of *C. gigas* when exposed to OsHV-1 under laboratory conditions

	OsHV-1				
	Spat 1	Spat 2	Juvenile 1	Juvenile 2	Adult
OsHV-1					
Spat 1	0.99 ± 0.39*				
Spat 2	1.06 ± 0.17*	0.65 ± 0.31*			
Juvenile 1	0.98 ± 0.18*	0.92 ± 0.11*	0.78 ± 0.33*		
Juvenile 2	0.55 ± 0.29	0.68 ± 0.23*	1.02 ± 0.12*	0.40 ± 0.21	
Adult	0.90 ± 0.30*	0.81 ± 0.24*	0.72 ± 0.23*	0.75 ± 0.24*	0.78 ± 0.49

Spat, oyster <2 cm/<5 g; Juvenile, oyster 2–5 cm/5–20 g; Adult, oyster >5 cm/>20 g

* Significantly different from 0

### Resistance to *V. aestuarianus* and to OsHV-1 infections are not correlated under laboratory conditions

Table 4 presents the genetic correlations of survival between *V. aestuarianus* and OsHV-1 infections within and between the three stages under laboratory conditions. No significant genetic correlation was observed between survival to viral infection and survival to bacterial infection. Excluding the genetic correlations between OsHV-1 and *V. aestuarianus* infections at Spat 1 and Spat 2 due to low levels of mortality with the bacteria, no particular trend was observed across life stages with genetic correlations ranging from −0.40 between OsHV-1 in adult and *V. aestuarianus* in Juvenile 1 to 0.57 between OsHV-1 in Spat 1 and *V. aestuarianus* in Juvenile 2 (Table 4).

**Table 4:** Genetic correlations of survival between diseases within or between stages under laboratory conditions

	*V. aestuarianus*				
	Spat 1	Spat 2	Juvenile 1	Juvenile 2	Adult
OsHV-1					
Spat 1	0.09 ± 0.51	0.63 ± 0.36	0.06 ± 0.42	0.57 ± 0.3	0.53 ± 0.45
Spat 2	0.20 ± 0.56	0.55 ± 0.43	−0.30 ± 0.46	0.10 ± 0.37	0.47 ± 0.42
Juvenile 1	1.03 ± 0.94	0.24 ± 0.43	0.14 ± 0.41	0.18 ± 0.36	0.41 ± 0.41
Juvenile 2	0.59 ± 0.70	−0.18 ± 0.44	0.02 ± 0.46	−0.02 ± 0.39	−0.01 ± 0.45
Adult	0.75 ± 0.69	0.44 ± 0.52	−0.40 ± 0.47	0.20 ± 0.42	0.42 ± 0.46

Spat, oyster <2 cm/<5 g; Juvenile, oyster 2–5 cm/5–20 g; Adult, oyster >5 cm/>20 g

### Mortality in field, pond, and raceway trials

Under field conditions, mortality rate was very high within 15 days post-planting, and mean mortality rate among the FSF reached 89%, ranging from 25 to 100% at 6 weeks post-planting. Nine families had a 100% mortality rate, and only six families had a mortality rate lower than 66%. The narrow sense heritability estimate of survival was equal to 2.15 and outside the parameter space (Table 5). A high load of OsHV-1 DNA copies was detected in all moribund oysters with a mean concentration of 4.8 × 10^9^ copies/µL for 25 ng of total DNA, whereas *V. aestuarianus* DNA was not detected in any of these oysters.

**Table 5:** Narrow sense heritability (in italics) and genetic correlations for survival between experimental infection challenges and field or raceway trials

	Stage	Field	Raceway	Experimental infection by *V. aestuarianus*				
		Spat	Juvenile	Spat 1	Spat 2	Juvenile 1	Juvenile 2	Adult
Field	Spat	*2.15* ± 0.43*	0.23 ± 0.33	0.41 ± 0.50	0.26 ± 0.35	−0.19 ± 0.39	0.19 ± 0.32	0.59 ± 0.32
Raceway	Juvenile	0.23 ± 0.33	*0.22* ± *0.13*	0.40 ± 0.57	0.47 ± 0.40	0.69 ± 0.27*	0.84 ± 0.24*	0.40 ± 0.44
Experimental infection by OsHV-1	Spat 1	0.88 ± 0.12*	0.35 ± 0.33					
	Spat 2	1.05 ± 0.07*	−0.06 ± 0.41					
	Juvenile 1	0.97 ± 0.09*	0.06 ± 0.39		See Table 4			
	Juvenile 2	0.77 ± 0.18*	−0.21 ± 0.43					
	Adult	0.78 ± 0.15*	0.02 ± 0.42					

Spat, oyster <2 cm/<5 g; Juvenile, oyster 2–5 cm/5–20 g; Adult, oyster >5 cm/>20 g

* Significantly different from 0

**Fig. 3:**
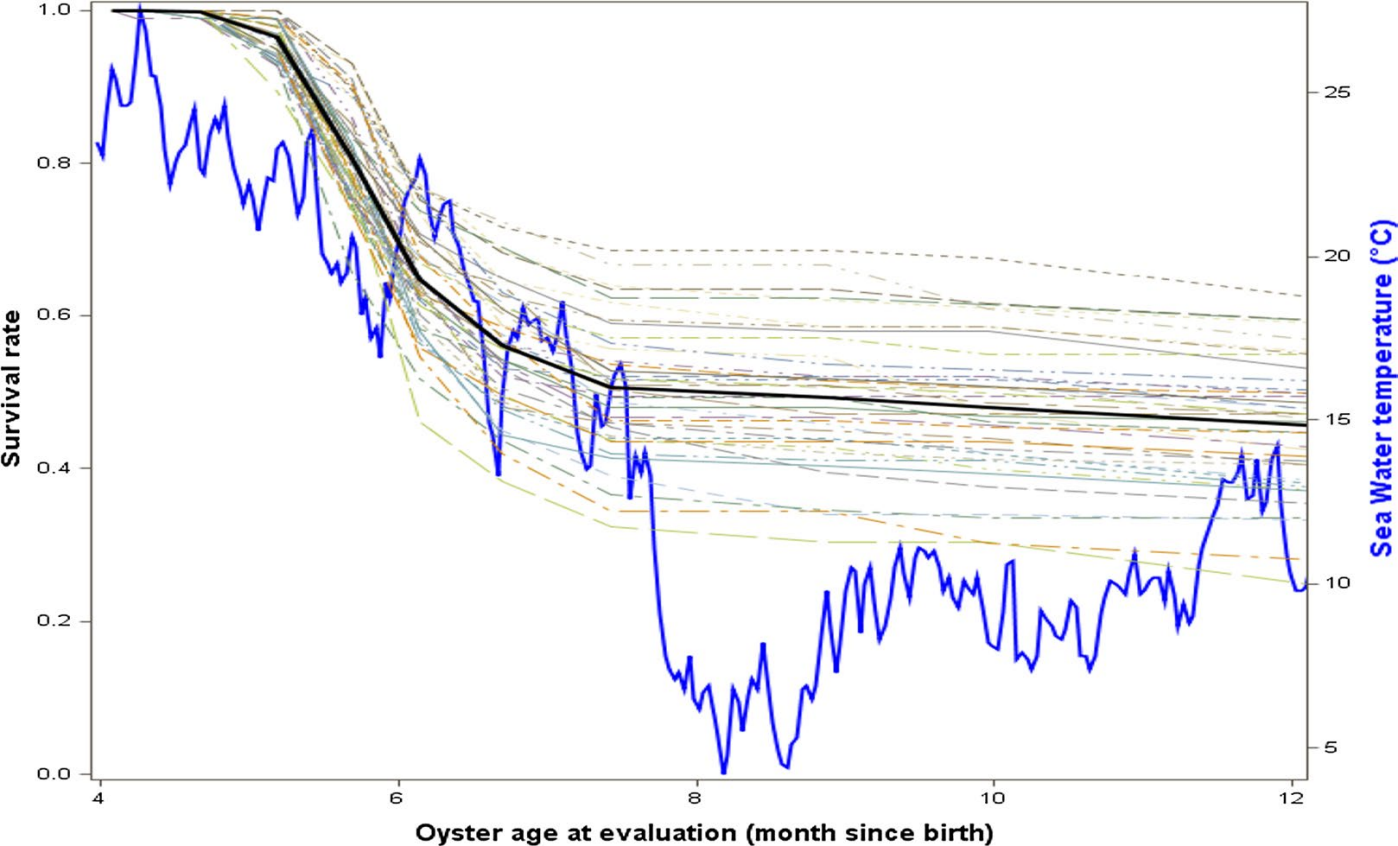
Spaghetti plot of the survival rate of the 40 FSF in pond conditions. Trial was conducted between July 2013 and February 2014. *Light lines* represent the survival rate of each FSF; *black line* represents the mean survival rate of all FSF; and *blue line* represents seawater temperature (°C)

In the experiments in the ponds, mortality began 1 month post-deployment (3.6% at day 34) (Fig. 3). Mortality rates reached 19.3, 35.1, 43.8, and 49.4% at days 49, 63, 80 and 102, respectively. Mortality rate stabilized during winter, when the temperature was below 13 °C, and was equal to 50.6 and 51.9% at days 146 and 179, respectively. The disease analyses performed on moribund oysters that were sampled during the mortality event demonstrated that neither OsHV-1 nor *V. aestuarianus* DNA was detected.

**Fig. 4:**
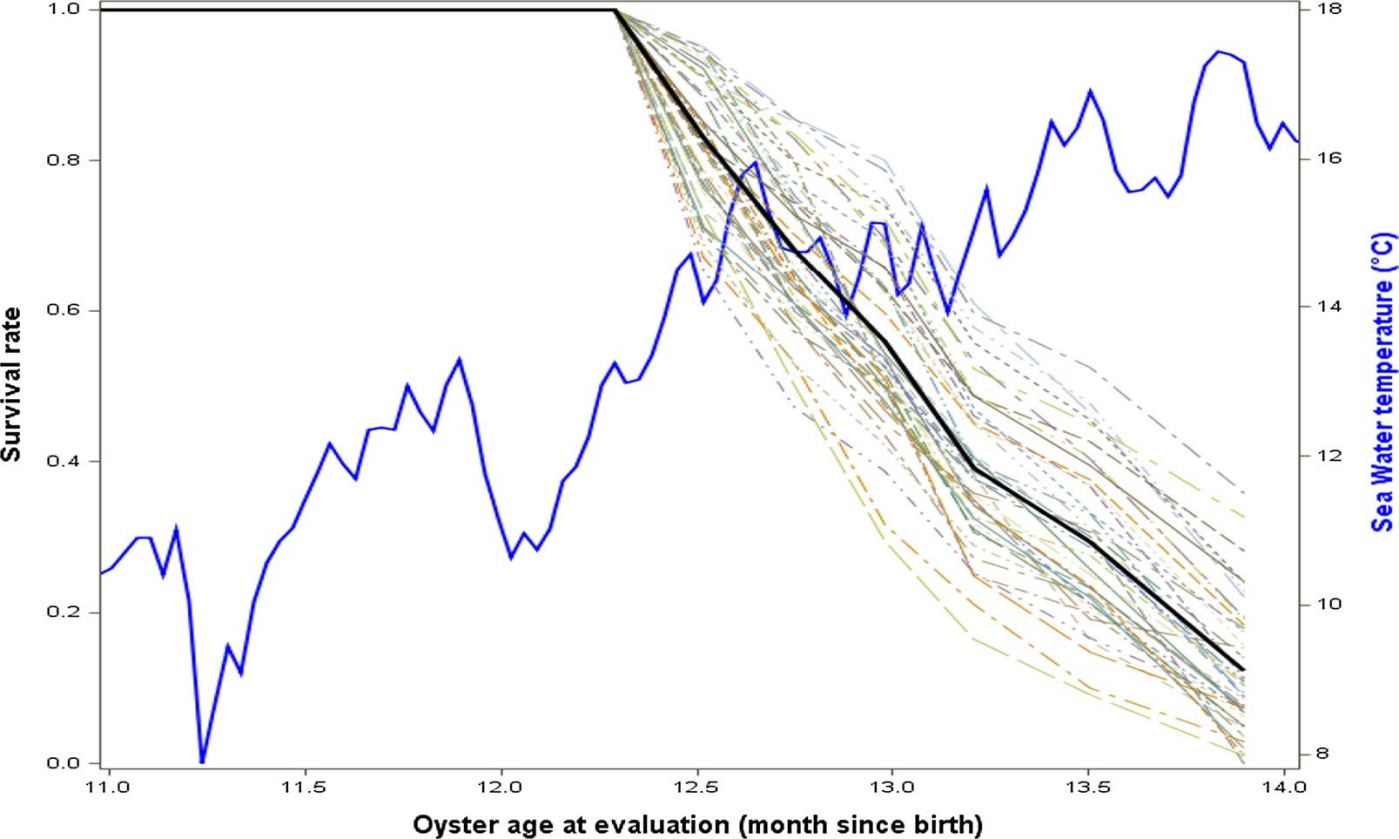
Spaghetti plot of the survival rate of the 40 FSF in raceway conditions. Trial was conducted between February 2014 and May 2014. *Light lines* represent the survival rate of each FSF; *black line* represents the mean survival rate of all FSF; and *blue line* represents seawater temperature (°C)

For juveniles tested in raceways, no mortality was recorded for the control throughout the experiment. For oysters that were temporally transferred into a pond during the 2 months before being tested in the raceways, no mortality was observed during winter (when the seawater temperature was below 13 °C). Onset of mortality occurred in April 2014 (Fig. 4), with mortality rates reaching 29, 9, and 10% in raceways 1, 2, and 3, respectively, and lasted 7 weeks. At the end of the experiment, mortality rates reached 92, 82, and 84% in raceways 1, 2, and 3, respectively, in May 2014. The narrow sense heritability of survival was equal to 0.22 ± 0.13 (Table 5). Large quantities of *V. aestuarianus* DNA were found (4.0 × 10^6^ copies/mL for 25 ng of total DNA) in all analyzed samples regardless of when sampling took place, from April to May. In contrast, OsHV-1 DNA was detected in some moribund oysters in May but always at a low copy number (<10^2^ copies/µL for 25 ng of total DNA) (Table 1).

### Mortalities in field or raceways experiments and in controlled disease challenges were correlated when the same pathogen was detected

Interestingly, genetic correlation estimates for survival were all positive and high between oysters tested in the field and oysters exposed to OsHV-1 under laboratory conditions, regardless of the life stage; they ranged from 0.77 to 1.05 (Table 5). To a lesser extent, genetic correlations were also high and positive between survival of juveniles in raceways and survival under *V. aestuarianus* challenges regardless of the life stage; they ranged from 0.40 to 0.84 (Table 5). Genetic correlations of 0.23 were found for survival between the field trial (OsHV-1 presence) and the raceway trial (*V. aestuarianus* presence) but they were not significant (*P* > 0.05) (Table 5).

## Discussion

Our study reveals for the first time that resistance to *V. aestuarianus* in *C. gigas* has a genetic basis and it also provides estimated heritabilities for resistance to a second pathogen, OsHV-1, in an oyster species. The challenges with *V. aestuarianus* demonstrated that the susceptibility to this pathogen was low for oysters in spat stages but increased in later life stages. Genetic variance of resistance to *V. aestuarianus* infection increased with life stages with a low heritability in spat (0.09 to 0.11) and moderate heritability in juvenile and adult stages (0.16 to 0.33) (Table 2). Our data suggest that genetic evaluation of *V. aestuarianus* resistance should be conducted at the juvenile or adult stages because of the increased mortality rate and higher heritability in these life stages.

The favorable genetic correlations between the laboratory and raceway experiments at the juvenile stage could allow selection under controlled conditions (Table 5). Furthermore, they suggest that the highly pathogenic strain (02/041) of *V. aestuarianus*, which was isolated during a mortality episode in adult oysters in 2002 [35] and used in our laboratory challenges, had a similar impact on FSF as the strain(s) of *V. aestuarianus* involved in the mortality observed in the raceways during the spring of 2014, which is consistent with the results reported in [47].

The second main result of our study is that resistance to OsHV-1 infection in spat, juveniles and adults has a strong genetic basis (Table 3). To date, narrow sense heritability estimates, ranging from 0.49 to 0.65, and realized heritability estimates, ranging from 0.34 to 0.63, for resistance to OsHV-1 infection were only available at the spat stage [48, 49]; these are in agreement with our results obtained from the OsHV-1 challenge under laboratory conditions. Among the FSF, a few displayed a high resistance to OsHV-1 infection, whereas a few displayed a high susceptibility to the disease regardless of the stage of development. Most of the FSF displayed increased resistance to OsHV-1 infection from the spat to adult stage, which highlights the importance of life stage for resistance to OsHV-1 infection in *C. gigas*. These results are supported by the high and positive genetic correlations between life stages (Table 3). According to the level of resistance, FSF are now characterized according to their level of resistance to OsHV-1 infection and can be used to further explore the physiological and immune mechanisms that underlie resistance to this infection.

Mortality occurring under the field conditions at Agnas was highly correlated with OsHV-1 infection under laboratory conditions (Table 5), which suggests that this pathogen has a major impact in the field and that the OsHV-1 challenge used in the laboratory effectively mimicked spat mortality events reported in the field. These results are consistent with those obtained for selected or unselected oysters infected with OsHV-1 [48, 50]. The estimated heritability for survival was higher than 1 when FSF were tested at Agnas (Table 5). This could be explained by (1) the high mortality rate (89%) among the FSF, which decreased the phenotypic variance and the precision of the estimation, and (2) the reduced number of sires and dams, which increased the probability of having an “inadmissible” estimated heritability higher than 1 [51].

The third main finding of our study was the lack of genetic correlations between resistance to OsHV-1 infection and resistance to *V. aestuarianus* infection (Table 4). Consequently, selection to improve resistance to OsHV-1 infection should neither increase nor decrease the resistance to *V. aestuarianus* infection. This also indicates that it would be possible to select for dual resistance in *C. gigas* as previously demonstrated with *Haplosporidium nelsoni* and *Perkinsus marinus* in *C. virginica* [52] and *Bonamia roughleyi* and *Martelia sydneyi* in *Saccostrea glomerata* [53], although these studies did not estimate the genetic correlations. Such findings were also obtained in several fish species with no genetic association between resistance to furunculosis and infectious salmon anemia (ISA) in *Salmo salar* [54] and between enteric redmouth disease, rainbow trout fry syndrome, and viral hemorrhagic septicemia in *Oncorhynchus mykiss* [55]. Meanwhile, another larger study revealed a genetic association that was significantly different from 0 between furunculosis and ISA resistance [56]. Thus, our finding that there is no genetic correlation between resistance to OsHV-1 infection and resistance to *V. aestuarianus* infection, which was based on a small number of sire families, may result from a sampling error, rather than indicate the absence of genetic relationship between resistance to OsHV-1 and *V. aestuarianus*.

Even with 36,760 oysters tested in the ten experiments under laboratory conditions and in the field, pond, and raceway conditions, the sample size used to estimate genetic parameters was relatively small, with only 20 HSF. Thus, the estimated heritabilities and genetic correlations may not be sufficiently accurate and should be taken with caution. Unfortunately, we could not increase the number of families because they were separated throughout the experiment. Consequently, our design was constrained due to a limited amount of available space for (1) larval production, (2) growing facilities to avoid mortality related to OsHV-1 and *V. aestuarianus* until genetic evaluation, in particular, until the adult stage, and more importantly, (3) running the experimental infections in the quarantine room from which no effluent returns into the sea. It would have been possible to increase the number of families by mixing the families at the fertilization step or the larval stage and then use parentage assignments as demonstrated for growth-related traits in *C. gigas* [21] and in *Mytilus galloprovincialis* [57]. However, the genetic evaluation of families is valid only if it is conducted in laboratory conditions and as long as all oysters, dead and surviving, within a challenge are sampled for pedigree assignments. Otherwise, the genetic variance for disease resistance would be confounded with the variance in reproductive success, which is high in *C. gigas* [58].

To avoid bias in the genetic evaluation of disease resistance, it is important to use disease-free oysters. For disease exposure under laboratory conditions, we used a cohabitation protocol between the FSF and oysters considered as non-infected and injected the infectious suspension into the adductor muscle. This protocol was assumed to be reproducible from spat to adult stages. Although injection protocols have been used for numerous studies with OsHV-1 and *V. aestuarianus* with demonstrated reproducibility [36, 38, 59, 60], these have only been used on a limited number of oysters for practical reasons. Studies have also demonstrated that the injection of *V. aestuarianus* induces high mortality rates, in particular when using strain 02/041 [34, 47, 59], and thus it is not adapted to genetic studies, since the variances of the resulting survival rates are then too low. Finally, this protocol is more biologically relevant because it better mimics a natural infection when compared to injection protocols that first impair oyster immune defenses.

Experimental challenges in the laboratory were complemented with deployments of the 40 FSF in three environments that are commonly used by oyster farmers: the field where oyster farms are located, raceways where oyster farmers stock their oysters several times during the growing phase, and ponds, which are mostly used in the Marennes-Oléron Bay and Pertuis Charentais sounds. In the field conditions, spat mortality reached 89% in 15 days and was associated with the detection of a large number of OsHV-1 DNA copies. In the raceway conditions, juvenile mortality reached 86% in 42 days and was also associated with the detection of a large number of *V. aestuarianus* DNA copies. Finally, spat deployed into the pond in July 2013 exhibited 50% mortality before winter (i.e. after 179 days), ranging from 31.4 to 69.5% among the FSF. Interestingly, the analyses on moribund oysters sampled during this last event did not reveal the presence of OsHV-1 DNA or *V. aestuarianus* DNA, and thus the causal factors of this mortality remain unknown. Ponds are commonly used by oyster producers to rear adults at low densities (5/m^2^) but not for spat. They may present unfavorable environmental conditions for spat, although they are assumed to limit the mortality rate due to OsHV-1 infection in *C. gigas* spat during the spring and summer [61]. Our results demonstrated that mortality can occur even if OsHV-1 and *V. aestuarianus* DNA are not detected, which suggests diverse causes of mortality and that other pathogenic agents may impact oyster survival under particular environmental conditions [62].

However, mortality related to *V. aestuarianus* was observed in the raceway experiment and it is likely that oysters were infected in the pond between December 2013 and February 2014. This hypothesis is not consistent with the absence of contamination of the oysters tested from the pond conditions. Such observations led us to consider the ecological niches of this bacterium and transmission mechanisms, as well as conditions that can control disease expression. While potential infection or transmission in the absence of disease expression has been demonstrated for OsHV-1 at low temperatures [63–65], it remains to be investigated for *V. aestuarianus*.

In our study, cross contamination was observed for four of the ten experiments conducted in the laboratory, which may have occurred through the seawater (Table 1). Indeed, filtration and UV-treated seawater probably removed or degraded most of the bacterial and viral particles, and the DNA that was detected was likely inactive or degraded. The absence of mortality in control tanks supported this statement, as well as the high genetic correlations for resistance to OsHV-1 between life stages (Table 3) and for resistance to *V. aestuarianus* between juvenile and adult stages (Table 2). The small number of viral DNA copies that were detected in some oysters during the bacterial challenge for Juvenile 2 and Adult could be explained by the coincidence of both experiments with the peak of mortality caused by OsHV-1, which occurs in May/June each year in the Marennes-Oléron Bay, where our facilities are located. This also supports the absence of viral DNA detected in oysters in the raceway conditions from February to April 2014, while a few copies of OsHV-1 DNA were found in some oysters in May 2014 (Table 1). Similarly, small to moderate copy numbers of *V. aestuarianus* DNA were found in some oysters during the OsHV-1 challenge for Juvenile 1 and Juvenile 2 (Table 1) during a period for which cases of mortality are regularly reported in the area. Finally, it was recently demonstrated that dual infections by OsHV-1 and *V. aestuarianus* led to very high mortality rates, even for oyster lines that were selected for higher resistance to OsHV-1 [66]. No such finding was observed in our study, which suggests that the estimated heritabilities and genetic correlations were for the pathogen tested.

## Conclusions

Our findings demonstrate that: (1) susceptibility to OsHV-1 infection decreased with age and growth, while susceptibility to *V. aestuarianus* infection increased according to the same factors; (2) genetic variances were high for resistance to OsHV-1 infection and low for resistance to *V. aestuarianus* infection; (3) genetic correlations between resistance to OsHV-1 infection and resistance to *V. aestuarianus* infection were not significantly different from 0; and (4) uncontrolled disease exposure and disease challenge under laboratory conditions presented significant positive genetic correlations when the same pathogenic agent was detected. Selective breeding to enhance the resistance to OsHV-1 infection could be achieved by selecting the highly resistant families at early stages without modifying the resistance to *V. aestuarianus* infection. Selection for dual resistance to OsHV-1 and *V. aestuarianus* infections in *C. gigas* may be possible to reduce the impact of these two major diseases by selecting oysters that have the highest breeding values for resistance to both diseases.

## Additional files

**Additional file 1: Figure S1.** Correlations between SAS and ASReml heritability estimates for survival in *C. gigas* when exposed to OsHV-1 or *V. aestuarianus* under controlled laboratory conditions in Spat 1, Spat 2, Juvenile 1, Juvenile 2 and Adult. *Black squares* represent *V. aestuarianus* challenge and black diamonds represent OsHV1 challenge. The data showed the heritabilities for survival estimated from either SAS or ASReml for each pathogen at each of the five experiments under controlled laboratory conditions: Spat 1, Spat 2, Juvenile 1, Juvenile 2 and Adult.

**Additional file 2: Table S1.** Variance components and narrow sense heritability for survival in *C. gigas* for each *V. aestuarianus* challenge under laboratory conditions (±SE). The data provided represent the variance components (sire, dam and phenotypic) and the narrow sense heritability for survival in *C. gigas* when exposed to *V. aestuarianus* under controlled laboratory conditions for each of the five experiments: Spat 1, Spat 2, Juvenile 1, Juvenile 2 and Adult.

**Additional file 3: Table S2.** Variance components and narrow sense heritability for survival in *C. gigas* for each OsHV-1 challenge under laboratory conditions (±SE). The data provided represent the variance components (sire, dam and phenotypic) and the narrow sense heritability for survival in *C. gigas* when exposed to OsHV-1 under controlled laboratory conditions for each of the five experiments: Spat 1, Spat 2, Juvenile 1, Juvenile 2 and Adult.

## References

[1] FAO. Food and Agriculture Organization of the United Nations. FishStatJ—software for fishery statistical time series. 2014. http://www.fao.org/fishery/statistics/software/fishstatj/en. Accessed 14 Dec 2016.

[2] Martin AG, Gérard A, Cochennec N, Langlade A. Selecting flat oysters, *Ostrea edulis*, for survival against the parasite *Bonamia ostreae*: assessment of the resistance of a first selected generation. In: Proceedings of the international conference bordeaux aquaculture ‘92. Bordeaux; 1993.

[3] Boudry P, Heurtebise S, Collet B, Cornette F, Gérard A (1998). Differentiation between populations of the Portuguese oyster, *Crassostrea angulata* (Lamark) and the Pacific oyster, *Crassostrea gigas* (Thunberg), revealed by mtDNA RFLP analysis. J Exp Mar Biol Ecol.

[4] Comps M, Duthoit JL (1976). Virus infection associated with ‘gill disease’ of the Portuguese Oyster *Crassostrea angulata* Lmk. C R Acad Sci.

[5] Grizel H, Héral M (1991). Introduction into France of the Japanese oyster (*Crassostrea gigas*). ICES J Mar Sci.

[6] Garcia C, Thébault A, Dégremont L, Arzul I, Miossec L, Robert M (2011). Ostreid herpesvirus 1 detection and relationship with *Crassostrea gigas* spat mortality in France between 1998 and 2006. Vet Res.

[7] Lacoste A, Jalabert F, Malham S, Cueff A, Gelebart F, Cordevant C (2001). A *Vibrio splendidus* strain is associated with summer mortality of juvenile oysters *Crassostrea gigas* in the Bay of Morlaix (North Brittany, France). Dis Aquat Organ.

[8] Segarra A, Pépin JF, Arzul I, Morga B, Faury N, Renault T (2010). Detection and description of a particular Ostreid herpesvirus 1 genotype associated with massive mortality outbreaks of Pacific oysters, *Crassostrea gigas*, in France in 2008. Virus Res.

[9] Martenot C, Fourour S, Oden E, Jouaux A, Travaillé E, Malas JP (2012). Detection of the OsHV-1µVar in the Pacific oyster *Crassostrea gigas* before 2008 in France and description of two new microvariants of the Ostreid Herpesvirus 1 (OsHV-1). Aquaculture.

[10] Pépin JF, Soletchnik P, Robert S. Mortalités massives de l’Huître creuse. Synthèse—Rapport final des études menées sur les mortalités de naissains d’huîtres creuses *C. gigas* sur le littoral charentais pour la période de 2007 à 2012. 2014. http://archimer.ifremer.fr/doc/00217/3285/ Accessed 14 Dec 2016.

[11] François C. Bilan 2014 du réseau Repamo—Réseau national de surveillance de la santé des mollusques marins. 2015. http://archimer.ifremer.fr/doc/00256/36691/35303.pdf. Accessed 14 Dec 2016.

[12] François C, Joly JP, Garcia C, Lupo C, Travers MA, Pépin JF, et al. Bilan 2012 du réseau REPAMO - Réseau national de surveillance de la santé des mollusques marins. 2013. http://archimer.ifremer.fr/doc/00143/25470/23625.pdf. Accessed 14 Dec 2016.

[13] François C, Joly JP, Garcia C, Lupo C, Travers MA, Tourbiez D, et al. Bilan 2013 du réseau Repamo—Réseau national de surveillance de la santé des mollusques marins. 2014. http://archimer.ifremer.fr/doc/00197/30798/29167.pdf. Accessed 14 Dec 2016.

[14] Labreuche Y, Lambert C, Soudant P, Boulo V, Huvet A, Nicolas JL (2006). Cellular and molecular hemocyte responses of the Pacific oyster, *Crassostrea gigas*, following bacterial infection with *Vibrio aestuarianus* strain 01/32. Microbes Infect.

[15] Normand J, Blin JL, Jouaux A (2014). Rearing practices identified as risk factors for ostreid herpesvirus 1 (OsHV-1) infection in Pacific oyster *Crassostrea gigas* spat. Dis Aquat Organ.

[16] Berthelin C, Kellner K, Mathieu M (2000). Storage metabolism in the Pacific oyster (*Crassostrea gigas*) in relation to summer mortalities and reproductive cycle (West Coast of France). Comp Biochem Physiol B Biochem Mol Biol.

[17] Pernet F, Barret J, Le Gall P, Corporeau C, Dégremont L, Lagarde F (2012). Mass mortalities of Pacific oysters *Crassostrea gigas* reflect infectious diseases and vary with farming practices in the Mediterranean Thau lagoon, France. Aquac Environ Interact.

[18] Boudry P, Burnell G, Allan G (2009). 3-genetic variation and selective breeding in hatchery-propagated molluscan shellfish. New technologies in aquaculture.

[19] Dégremont L, Garcia C, Allen SK (2015). Genetic improvement for disease resistance in oysters: a review. J Invertebr Pathol.

[20] Wang Q, Li Q, Kong L, Yu R (2012). Response to selection for fast growth in the second generation of Pacific oyster (*Crassostrea gigas*). J Ocean Univ China.

[21] Kong N, Li Q, Yu H, Kong LF (2015). Heritability estimates for growth-related traits in the Pacific oyster (*Crassostrea gigas*) using a molecular pedigree. Aquac Res.

[22] Li Q, Wang Q, Liu S, Kong L (2011). Selection response and realized heritability for growth in three stocks of the Pacific oyster *Crassostrea gigas*. Fish Sci.

[23] Jiang Q, Li Q, Yu H, Kong LF (2013). Genetic and epigenetic variation in mass selection populations of Pacific oyster *Crassostrea gigas*. Genes Genomics.

[24] Langdon C, Evans F, Jacobson D, Blouin M (2003). Yields of cultured Pacific oysters *Crassostrea gigas* Thunberg improved after one generation of selection. Aquaculture.

[25] Evans S, Camara MD, Langdon CJ (2009). Heritability of shell pigmentation in the Pacific oyster, *Crassostrea gigas*. Aquaculture.

[26] Clegg TA, Morrissey T, Geoghegan F, Martin SW, Lyons K, Ashe S (2014). Risk factors associated with increased mortality of farmed Pacific oysters in Ireland during 2011. Prev Vet Med.

[27] Paul-Pont I, Evans O, Dhand NK, Rubio A, Coad P, Whittington RJ (2014). Descriptive epidemiology of mass mortality due to Ostreid herpesvirus-1 (OsHV-1) in commercially farmed Pacific oysters (*Crassostrea gigas*) in the Hawkesbury River estuary, Australia. Aquaculture..

[28] Dégremont L (2011). Evidence of herpesvirus (OsHV-1) resistance in juvenile *Crassostrea gigas* selected for high resistance to the summer mortality phenomenon. Aquaculture.

[29] Segarra A, Baillon L, Tourbiez D, Benabdelmouna A, Faury N, Bourgougnon N (2014). Ostreid herpesvirus type 1 replication and host response in adult Pacific oysters, *Crassostrea gigas*. Vet Res.

[30] Whittington RJ, Dhand NK, Evans O, Paul-Pont I (2015). Further observations on the influence of husbandry practices on OsHV-1 μVar mortality in Pacific oysters *Crassostrea gigas*: age, cultivation structures and growing height. Aquaculture.

[31] Dégremont L (2013). Size and genotype affect resistance to mortality caused by OsHV-1 in *Crassostrea gigas*. Aquaculture.

[32] Petton B, Boudry P, Alunno-Bruscia M, Pernet F (2015). Factors influencing disease-induced mortality of Pacific oysters *Crassostrea gigas*. Aquac Environ Interact.

[33] De Decker S. A study of the Vibrio pathogens virulence in relation to the variability of the oyster’s response. In: Workshop of the University of Poitiers. Poitiers; 2007.

[34] Azéma P, Travers MA, De Lorgeril J, Tourbiez D, Dégremont L (2015). Can selection for resistance to OsHV-1 infection modify susceptibility to *Vibrio aestuarianus* infection in *Crassostrea gigas*? First insights from experimental challenges using primary and successive exposures. Vet Res.

[35] Saulnier D, De Decker S, Haffner P, Cobret L, Robert M, Garcia C (2010). A large-scale epidemiological study to identify bacteria pathogenic to Pacific oyster *Crassostrea gigas* and correlation between virulence and metalloprotease-like activity. Microb Ecol.

[36] Schikorski D, Renault T, Saulnier D, Faury N, Moreau P, Pépin JF (2011). Experimental infection of Pacific oyster *Crassostrea gigas* spat by ostreid herpesvirus 1: demonstration of oyster spat susceptibility. Vet Res.

[37] Suquet M, de Kermoysan G, Araya RG, Queau I, Lebrun L, Le Souchu P (2009). Anesthesia in Pacific oyster, *Crassostrea gigas*. Aquat Living Resour.

[38] Schikorski D, Faury N, Pépin JF, Saulnier D, Tourbiez D, Renault T (2011). Experimental ostreid herpesvirus 1 infection of the Pacific oyster *Crassostrea gigas*: kinetics of virus DNA detection by q-PCR in seawater and in oyster samples. Virus Res.

[39] De Decker S, Saulnier D (2011). Vibriosis induced by experimental cohabitation in *Crassostrea gigas*: evidence of early infection and down-expression of immune-related genes. Fish Shellfish Immunol.

[40] Webb SC, Fidler A, Renault T (2007). Primers for PCR-based detection of ostreid herpes virus-1 (OsHV-1): application in a survey of New Zealand molluscs. Aquaculture.

[41] Saulnier D, De Decker S, Haffner P (2009). Real-time PCR assay for rapid detection and quantification of *Vibrio aestuarianus* in oyster and seawater: a useful tool for epidemiologic studies. J Microbiol Methods.

[42] Ødegård J, Baranski M, Gjerde B, Gjedrem T (2011). Methodology for genetic evaluation of disease resistance in aquaculture species: challenges and future prospects. Aquac Res.

[43] SAS Institute Inc. SAS/STAT^®^ 12.1 User’s Guide. Cary, NC: SAS Institute Inc; 2012. https://support.sas.com/documentation/onlinedoc/stat/121/glimmix.pdf.

[44] Gilmour AR, Gogel BJ, Cullis BR, Welham SJ, Thompson R (2015). ASReml user guide release 4.1 structural specification.

[45] Lynch M, Walsh B (1998). Genetics and analysis of quantitative traits.

[46] Gilmour AR, Anderson RD, Rae AL (1985). The analysis of binomial data by a generalized linear mixed model. Biometrika.

[47] Goudenège D, Travers MA, Lemire A, Petton B, Haffner P, Labreuche Y (2015). A single regulatory gene is sufficient to alter *Vibrio aestuarianus* pathogenicity in oysters. Environ Microbiol.

[48] Dégremont L, Lamy JB, Pépin JF, Travers MA, Renault T (2015). New insight for the genetic evaluation of resistance to ostreid herpesvirus infection, a worldwide Disease, in *Crassostrea gigas*. PLoS One.

[49] Dégremont L, Nourry M, Maurouard E (2015). Mass selection for survival and resistance to OsHV-1 infection in *Crassostrea gigas* spat in field conditions: response to selection after four generations. Aquaculture.

[50] Dégremont L, Soletchnik P, Boudry P (2010). Summer mortality of selected juvenile Pacific oyster *Crassostrea gigas* under laboratory conditions and in comparison with field performance. J Shellfish Res.

[51] Prabhakaran VT, Jain JP (1987). Probability of inadmissible estimates of heritability from regression and half-sib analyses. Biometr J.

[52] Ragone Calvo LMR, Calvo GW, Burreson EM (2003). Dual disease resistance in a selectively bred eastern oyster, *Crassostrea virginica*, strain tested in Chesapeake Bay. Aquaculture.

[53] Dove MC, Nell JA, O’Connor WA (2013). Evaluation of the progeny of the fourth-generation Sydney rock oyster *Saccostrea glomerata* (Gould, 1850) breeding lines for resistance to QX disease (*Marteilia sydneyi*) and winter mortality (*Bonamia roughleyi*). Aquacult Res.

[54] Gjerde B, Evensen Ø, Bentsen HB, Storset A (2009). Genetic (co)variation of vaccine injuries and innate resistance to furunculosis (*Aeromonas salmonicida*) and infectious salmon anaemia (ISA) in Atlantic salmon (*Salmo salar*). Aquaculture.

[55] Henryon M, Berg P, Olesen NJ, Kjær TE, Slierendrecht WJ, Jokumsen A (2005). Selective breeding provides an approach to increase resistance of rainbow trout (*Onchorhynchus mykiss*) to the diseases, enteric redmouth disease, rainbow trout fry syndrome, and viral haemorrhagic septicaemia. Aquaculture.

[56] Ødegård J, Olesen I, Gjerde B, Klemetsdal G (2007). Positive genetic correlation between resistance to bacterial (furunculosis) and viral (infectious salmon anaemia) diseases in farmed Atlantic salmon (*Salmo salar*). Aquaculture.

[57] Nguyen TTT, Hayes BJ, Ingram BA (2014). Genetic parameters and response to selection in blue mussel (*Mytilus galloprovincialis*) using a SNP-based pedigree. Aquaculture.

[58] Boudry P, Collet B, Cornette F, Hervouet V, Bonhomme F (2002). High variance in reproductive success of the Pacific oyster (*Crassostrea gigas*, Thunberg) revealed by microsatellite-based parentage analysis of multifactorial crosses. Aquaculture.

[59] Meng J, Zhang L, Huang B, Li L, Zhang G (2015). Comparative analysis of oyster (*Crassostrea gigas*) immune responses under challenge by different Vibrio strains and conditions. Molluscan Res.

[60] Paul-Pont I, Evans O, Dhand NK, Whittington RJ (2015). Experimental infections of Pacific oyster *Crassostrea gigas* using the Australian ostreid herpesvirus-1 (OsHV-1) mu Var strain. Dis Aquat Organ.

[61] Dégremont L, Benabdelmouna A (2014). Mortality associated with OsHV-1 in spat *Crassostrea gigas*: role of wild-caught spat in the horizontal transmission of the disease. Aquac Int.

[62] Petton B, Bruto M, James A, Labreuche Y, Alunno-Bruscia M, Le Roux F (2015). *Crassostrea gigas* mortality in France: the usual suspect, a herpes virus, may not be the killer in this polymicrobial opportunistic disease. Front Microbiol.

[63] Pernet F, Tamayo D, Petton B (2015). Influence of low temperatures on the survival of the Pacific oyster (*Crassostrea gigas*) infected with ostreid herpes virus type 1. Aquaculture.

[64] Renault T, Bouquet AL, Maurice JT, Lupo C, Blachier P (2014). Ostreid herpesvirus 1 infection among Pacific oyster (*Crassostrea gigas*) spat: relevance of water temperature to virus replication and circulation prior to the onset of mortality. Appl Environ Microbiol.

[65] Dégremont L, Guyader T, Tourbiez D, Pépin JF (2013). Is horizontal transmission of the Ostreid herpesvirus OsHV-1 in *Crassostrea gigas* affected by unselected or selected survival status in adults to juveniles?. Aquaculture.

[66] Azéma P, Travers MA, Benabdelmouna A, Dégremont L (2016). Single or dual experimental infections with *Vibrio aestuarianus* and OsHV-1 in diploid and triploid *Crassostrea gigas* at the spat, juvenile and adult stages. J Invertebr Pathol.

