# Supplementary material for: Genetic parameters of resistance to Vibrio aestuarianus, and OsHV-1 infections in the Pacific oyster, Crassostrea gigas, at three different life stages
Source: Genet Sel Evol. 2017 Feb 15;49:23. doi: 10.1186/s12711-017-0297-2 (PMC5311879; doi:10.1186/s12711-017-0297-2)
Supplement: Supplementary file 1 — Additional file 1: Figure S1. Correlations between SAS and ASReml heritability estimates for survival in C. gigas when exposed to OsHV-1 or V. aestuarianus under controlled laboratory conditions in Spat 1, Spat 2, Juvenile 1, Juvenile 2 and Adult. Black squares represent V. aestuarianus challenge and black diamonds represent OsHV1 challenge. The data showed the heritabilities for survival estimated from either SAS or ASReml for each pathogen at each of the five experiments under controlled laboratory conditions: Spat 1, Spat 2, Juvenile 1, Juvenile 2 and Adult. [file 12711_2017_297_MOESM1_ESM.docx]

**Figure S1: Correlations between SAS and ASReml heritability estimates for survival in *C. gigas* when exposed to OsHV-1 or *V. aestuarianus* under controlled laboratory conditions in Spat1, Spat2, Juvenile1, Juvenile2 and Adult. Black squares represents *V. aestuarianus* challenge and black diamonds represents OsHV1 challenge.**
