# Supplementary material for: Genetic parameters of resistance to Vibrio aestuarianus, and OsHV-1 infections in the Pacific oyster, Crassostrea gigas, at three different life stages
Source: Genet Sel Evol. 2017 Feb 15;49:23. doi: 10.1186/s12711-017-0297-2 (PMC5311879; doi:10.1186/s12711-017-0297-2)
Supplement: Supplementary file 2 — Additional file 2: Table S1. Variance components and narrow sense heritability for survival in C. gigas for each V. aestuarianus challenge under laboratory conditions (±SE). The data provided represent the variance components (sire, dam and phenotypic) and the narrow sense heritability for survival in C. gigas when exposed to V. aestuarianus under controlled laboratory conditions for each of the five experiments: Spat 1, Spat 2, Juvenile 1, Juvenile 2 and Adult. [file 12711_2017_297_MOESM2_ESM.docx]

Table S1: Variance components and narrow sense heritabilities for survival in *C. gigas* for each *V.aestuarianus* challenge in the laboratory (±SE)

| Variance | Spat 1 | Spat 2 | Juvenile 1 | Juvenile 2 | Adult |
| --- | --- | --- | --- | --- | --- |
| V_sire_ | 0.08 ± 0.09 | 0.09 ± 0.06 | 0.25 ± 0.17 | 0.14 ± 0.08 | 0.31 ± 0.25 |
| V_dam_ | -0.01 ± -0.12 | 0.07 ± 0.05 | 0.35 ± 0.15 | 0.10 ± 0.05 | 0.26 ± 0.23 |
| V_phenotypic_ | 3.35 ± 0.09 | 3.45 ± 0.07 | 3.89 ± 0.18 | 3.50 ± 0.18 | 3.86 ± 0.25 |
| h²_narrow sense_ | 0.09 ± 0.10 | 0.11 ± 0.07 | 0.26 ± 0.17 | 0.16 ± 0.09 | 0.33 ± 0.25 |
