# Supplementary material for: Genetic parameters of resistance to Vibrio aestuarianus, and OsHV-1 infections in the Pacific oyster, Crassostrea gigas, at three different life stages
Source: Genet Sel Evol. 2017 Feb 15;49:23. doi: 10.1186/s12711-017-0297-2 (PMC5311879; doi:10.1186/s12711-017-0297-2)
Supplement: Supplementary file 3 — Additional file 3: Table S2. Variance components and narrow sense heritability for survival in C. gigas for each OsHV-1 challenge under laboratory conditions (±SE). The data provided represent the variance components (sire, dam and phenotypic) and the narrow sense heritability for survival in C. gigas when exposed to OsHV-1 under controlled laboratory conditions for each of the five experiments: Spat 1, Spat 2, Juvenile 1, Juvenile 2 and Adult. [file 12711_2017_297_MOESM3_ESM.docx]

Table S2: Variance components and narrow sense heritabilities for survival in *C. gigas* for each OsHV-1 challenge in the laboratory (±SE)

| Variance | Spat 1 | Spat 2 | Juvenile 1 | Juvenile 2 | Adult |
| --- | --- | --- | --- | --- | --- |
| V_sire_ | 1.41 ± 0.69 | 0.78 ± 0.42 | 0.99 ± 0.50 | 0.41 ± 0.23 | 1.14 ± 0.80 |
| V_dam_ | 0.97 ± 0.40 | 0.73 ± 0.27 | 0.79 ± 0.30 | 0.42 ± 0.16 | 1.44 ± 0.69 |
| V_phenotypic_ | 5.67 ± 0.70 | 4.80 ± 0.42 | 5.07 ± 0.50 | 4.12 ± 0.23 | 5.88 ± 0.83 |
| h²_narrow sense_ | 0.99 ± 0.39 | 0.65 ± 0.31 | 0.78 ± 0.33 | 0.40 ± 0.21 | 0.78 ± 0.78 |
